# Taking Stock of Biodiversity to Stem Its Rapid Decline

**DOI:** 10.1371/journal.pbio.0020413

**Published:** 2004-10-26

Far more species exist in the fossil record than inhabit Earth today. An estimated 94% of all bird species that ever lived, for example, are now extinct. So why is species extinction of urgent concern today? Though species come and go over evolutionary time, mass extinctions are relatively rare. Biologists believe they have occurred only five times, arising from relatively short-lived cataclysmic natural forces like astronomical or volcanic events. We are now on the brink of the Sixth Great Extinction, and we humans are largely to blame. For thousands of years, humans have retooled the landscape, an endeavor that has rarely coincided with the life history needs of local flora and fauna: over 150 bird species alone have vanished since 1500.

As our capacity to alter the landscape has mushroomed, species have started disappearing faster than biologists can identify and document them. Mindful of this crisis, nearly 200 countries (under the Convention on Biological Diversity, or CBD) agreed to staunch the loss of biodiversity by 2010, with the European Union raising the bar to *halt* biodiversity loss by that time. To meet this goal, biologists need reliable metrics to monitor global trends in biodiversity. Stuart Butchart et al. describe a new model for generating such indices to measure trends in extinction risk for complete classes of organisms, starting with the world's 10,000 bird species. Their “Red List Index” measures changes in overall extinction risk over time for all bird species worldwide. Similar indices are already being developed to track other groups, including mammals and amphibians, and in the future will hopefully be developed for some plant and invertebrate groups.

In 2002, the CBD proposed that efforts to monitor global trends in biodiversity start by developing indicators to evaluate trends in biodiversity components, such as ecosystems and habitats, abundance and distribution of selected species, and change in threat status of species. Butchart et al. focus on evaluating trends in changes in threat status (extinction risk), relying on categories developed by the World Conservation Union (IUCN) Red List. Species are placed in categories on the Red List ranging from extinct to “least concern,” according to criteria that take into account their population size, population trends, and range size. Thousands of scientists from around the world feed these assessments, which have been widely used to measure the degree of degradation of biodiversity.[Fig pbio-0020413-g001]

**Figure pbio-0020413-g001:**
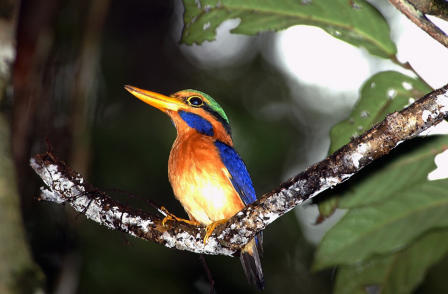
Rufous-collared Kingfisher: Deforestation threatens its survival (Photo: Jakob Wijkema)

To use the Red List to track biodiversity trends over time, Butchart et al. collected data from four complete assessments of the world's birds over sixteen years, supplemented by other sources. The number of threatened and near threatened species increased from 1,664 species in 1988 to 1,990 species in 2004, but many species moved between categories. To calculate the real net increase in extinction risk for the world's birds over this time, the authors first identified reasons for these category switches to remove biases introduced by factors irrelevant to genuine changes in species status; category changes owing to better knowledge, for example, do not reflect real changes in conservation status. They also accounted for time lags between status changes, and category changes owing to delays in knowledge becoming available to Red List assessors.

The authors argue that the Red List Index provides a simple measure of trends in the status of avian species worldwide, in terms of their overall extinction risk. Overall, the index shows “a steady and continuing deterioration in the threat status of the world's birds between 1988 and 2004.” Though the extinction risk has improved for some species, it has deteriorated for others, with “particularly steep declines” in recent years for Asian birds—resulting from massive deforestation in Indonesia—and for seabirds such as albatrosses and petrels, which drown on the hooks of commercial long-line fisheries.

Butchart et al. argue that Red List Indices complement indicators based on population trends, because although the indices show coarser temporal resolution, they have much better geographic representation; they're based on nearly all species in a group worldwide rather than on a potentially biased subset. Both types of species-based indicators show finer ecological resolution in tracking biodiversity loss than indicators like habitat or biome trends. Thus, the Red List Index provides a baseline for tracking progress toward the 2010 target. But having a reliable indicator is only the first step. Without an international commitment to halt the advancing extinction crisis, biodiversity will continue to decrease. The United States is the only industrialized nation that has not signed on to this effort.

